# Identification of Ameloblastin as an Amyloid Precursor Protein of Amyloid-Producing Ameloblastoma in Dogs and Cats

**DOI:** 10.3390/vetsci10020166

**Published:** 2023-02-20

**Authors:** Niki Sedghi Masoud, Susumu Iwaide, Yoshiyuki Itoh, Miki Hisada, Tomoyuki Harada, Tomoaki Murakami

**Affiliations:** 1 Laboratory of Veterinary Toxicology, Tokyo University of Agriculture and Technology, Tokyo 183-8509, Japan; 2 Smart-Core-Facility Promotion Organization, Tokyo University of Agriculture and Technology, Tokyo 183-8509, Japan; 3 FUJIFILM VET Systems Co., Ltd., Tokyo 185-0013, Japan

**Keywords:** amyloidosis, ameloblastin, tumor

## Abstract

**Simple Summary:**

Amyloid-producing ameloblastoma (APAB) is a rare type of odontogenic tumor found in cats and dogs. This study was conducted to explore this condition further by using proteomic analysis based on mass spectrometry in dogs and cats and identified ameloblastin as an amyloid precursor protein. This study also showed that the N-terminal region of ameloblastin may be involved in the amyloidogenesis.

**Abstract:**

Amyloid-producing ameloblastoma (APAB) is characterized by abundant amyloid deposits in ameloblastoma, but the amyloid precursor protein is unknown. To explore this, we conducted histopathologic and proteomic analyses on formalin-fixed and paraffin-embedded samples from five cases of APAB (three dogs and two cats). Histologically, the samples exhibited a proliferation of the odontogenic epithelium, with moderate to severe interstitial amyloid deposits. By using Congo red and polarized light, the amyloid deposits were found to show characteristic birefringence. Amyloid deposits were dissected from tissue sections and analyzed by LC/MS/MS, and high levels of ameloblastin were detected in all tissues. Mass spectrometry also revealed that the N-terminal region of ameloblastin is predominantly present in amyloid deposits. Immunohistochemistry was performed using two anti-ameloblastin (N terminal, middle region) antibodies and showed that amyloid deposits were positive for ameloblastin N terminal but negative for ameloblastin middle region. These results suggest that ameloblastin is the amyloid precursor protein of APABs in dogs and cats, and the N-terminal region may be involved in the amyloidogenesis of ameloblastin.

## 1. Introduction

Amyloidosis is a group of rare and diverse disorders where abnormal proteins are deposited in tissues [1]. These proteins form amyloid, which is a cluster of protein layers arranged in a cross β-sheet structure. When these layers stack together, they form amyloid fibrils. These characteristic structures contribute to specific binding to Congo red and thioflavin dyes. Amyloidosis in humans has been classified into 42 types based on their precursor proteins, out of which 14 types lead to systemic deposits, 24 types develop only in local amyloid, and 4 types can occur in both forms. However, in animals, only 11 amyloidoses have been biochemically characterized. It seems that many more animal amyloidoses will be discovered in the future. Amyloid deposits can be in systemic organs (systemic amyloidosis) or restricted to specific organs (localized amyloidosis). If the precursor protein is derived from serum, it will be systemic amyloidosis; if produced locally, it will result in localized amyloidosis [2]. Localized amyloidosis is a condition commonly found in humans, which affects the endocrine organs and is associated with neoplastic conditions. Cerebral amyloidosis, observed in Alzheimer’s disease, is another type of localized amyloidosis in humans [3,4]. In animals, localized amyloidosis has been linked to neoplastic conditions, such as pancreatic endocrine tumor [5], medullary thyroid carcinoma [6], ameloblastoma [7], mammary carcinoma [8], intestinal carcinoid [9], extramedullary plasmacytoma [10,11], and myeloma [12]. Localized amyloid deposition in odontogenic tumors has been reported in cats, dogs, a goat, a prairie dog (*Cynomys ludovicianus*), and a Bengal tiger (*Panthera tigris tigris*) [13,14,15,16,17,18,19,20]. This pathological condition in domestic animals had been named amyloid-producing odontogenic tumor but was renamed to amyloid-producing ameloblastoma (APAB), as it generally fulfills the criteria for ameloblastoma [21]. This tumor in dogs and cats is restricted to the gingiva of the mandible and maxilla and is typically located in the middle of the bone. Although APABs occasionally recur following surgical excision, it might be difficult to perform complete excision owing to their invasive feature and involvement in the bone tissue. As a result, recurrence after resection has been reported [22]. Abundant amyloid deposits are a significant characteristic of this tumor [19,20,23,24]. While APAB was once thought to be similar to calcifying epithelial odontogenic tumor (CEOT) in humans due to the similarity in amyloid deposition, the current agreement is that there is no real equivalent of APAB in CEOT [24,25]. In human CEOT, odontogenic ameloblast-associated protein has been identified as an amyloid precursor protein [2]. Although several immunohistochemistry-based studies have shown that ameloblastin [17,26], sheathlin, and amelogenin [17] are present in amyloid deposits, the amyloid precursor proteins of APAB have not yet been precisely identified. In the present study, we performed mass-spectrometry-based proteomic analysis of three dogs and two cats with APAB and identified ameloblastin as an amyloid precursor protein.

## 2. Materials and Methods

### 2.1. Case Information

Formalin-fixed and paraffin-embedded (FFPE) specimens of mandibular gingival masses from three dogs and maxillary gingival masses from two cats were subjected to analysis. Detailed case information is presented in Table 1. All dogs and cats were reared in households. The masses were harvested at private veterinary hospitals between 2018 and 2021, fixed in formalin, and sent to the authors’ laboratory for pathological testing.

### 2.2. Histopathological Analysis

Formalin-fixed tissues were embedded in paraffin wax, cut into 3 µm sections, and stained with hematoxylin and eosin and Congo red. Histopathological diagnosis of APABs was made by two Japanese-College-of-Veterinary-Pathologists-licensed veterinary pathologists (Harada and Murakami). Amyloid deposits were identified as yellow to green birefringent materials of Congo-red-stained specimens under polarized light.

### 2.3. Mass-Spectrometry-Based Proteomic Analysis

FFPE specimens of all cases were cut into 8 µm sections, put on PEN membrane glass slides (Thermo Fisher Scientific, Tokyo, Japan), and stained with Congo red. Dissection, solubilization and tryptic digestion of Congo-red-positive amyloid lesions were performed, as described previously [8]. Liquid chromatography/tandem mass spectrometry (LC/MS/MS) of digested and MS/MS data acquisition were performed as described previously [8].

The MS/MS data were collated with the theoretical fragmentation patterns of tryptic peptide sequences of proteins in the NCBI database using Mascot Server (Matrix Science Inc., Boston, MA, USA). Because the exact sequence information for the feline ameloblastin was not yet registered (Figure S1), Mascot analysis of all samples was conducted using the canine database (accession: GCF_000002285.3). In silico proteolytic enzyme “semiTrypsin” was selected to identify non-tryptic peptides. Statistically significant peptides (*p* < 0.05) were extracted using Mascot’s probability-based scoring algorithm.

### 2.4. Immunohistochemistry (IHC)

IHC was performed with the following primary antibodies: anti-N-terminal region (immunogen: 21-120/447) of human ameloblastin (orb155652, Biorbyt, Cambridge, UK), anti-middle region (exact information of immunogen unavailable) of human ameloblastin (PA5-70532, Invitrogen, Waltham, MA, USA), and anti-human odontogenic ameloblast-associated protein (16509-1-AP, Proteintech, Rosemont, IL, USA). For antigen retrieval, specimens were autoclaved in deionized water at 121 °C for 20 min before reaction with the primary antibodies. Horseradish-peroxidase-labeled polymer anti-rabbit immunoglobulin antibody (Dako, Santa clara, CA, USA) was used as a secondary antibody. A diaminobenzidine-4HCl substrate kit (Liquid DAB + Substrate Chromogen System, Dako) and hematoxylin were used for color development and counterstaining, respectively.

## 3. Results

### 3.1. Histopathology

**Figure 1:**
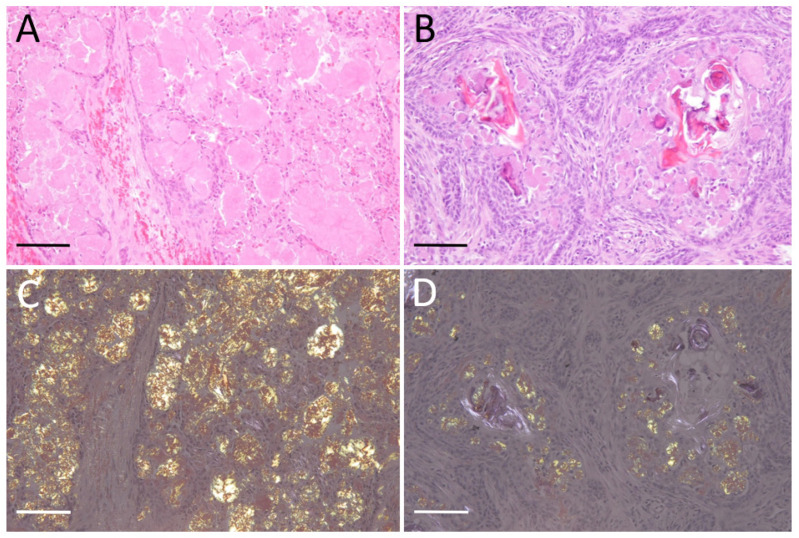
Amyloid-producing ameloblastoma (APAB); (**A**) APAB; dog No. 3. Cystic proliferation of odontogenic epithelium and amyloid deposits in island and stroma. H&E stain. (**B**) APAB; cat No. 2. Left maxillary mass, severe amyloid deposits in interstitium and tumor epithelial cells. H&E stain. (**C**) APAB; dog No. 3. Amyloid deposits show yellow-green birefringence under polarized light. Congo red stain. (**D**) APAB; cat No. 2. Congo red stain. Scale bars: 200 μm.

Histologically, all cases showed variable microscopic organization, including irregular nests, islands, and strands of proliferating odontogenic epithelium, which were supported by varying amounts of fibrous stroma. Tumor nests and islands are disrupted by moderate to severe amorphous extracellular amyloid deposits (Figure 1A,B). Multiple homogeneous eosinophilic materials were stained with Congo red and showed green to yellow birefringence under polarized light, indicative of amyloid (Figure 1C,D). Therefore, all cases were diagnosed as APAB.

### 3.2. Proteomic Analysis

Table 2 shows the proteomic analysis results, illustrating that ameloblastin was detected at a high level in all samples. Keratin 14 and keratin 5, tumor markers for ameloblastoma, were also detected in four of five cases. Apolipoprotein A-IV, apolipoprotein A-I, vitronectin, and clusterin were detected as amyloid signature proteins [27], proving that the dissected tissue extracts contain amyloid. Odontogenic ameloblast-associated protein, the amyloid precursor protein of human CEOT, was not detected. In addition, neither sheathlin nor amelogenin, which were immunohistochemically detected in animal APABs in previous studies [17,26], were detected in all cases in this study.

**Figure 2:**
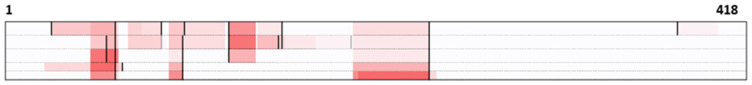
Detected peptides by mass spectrometry in five cases; each row of dog cases (1–3) and cat cases (1, 2) is colored from top to bottom based on the total number of peptides detected in five cases. Warmer colors indicate a higher number of peptides detected. The maximum number of detections (shown in red) was 10, 13, 14, 4, and 4, respectively. Black bars indicate non-tryptic truncation sites.

To verify which regions of ameloblastin constitute amyloid, the peptides detected by mass spectrometry were mapped (Figure 2). Based on the analysis of ameloblastin-derived peptides, most peptides were derived from the N terminal to central regions. In contrast, few C-terminal-derived peptides were detected. Within the N-terminal to center regions, several non-tryptic truncations were observed (Figure 2).

### 3.3. Immunohistochemistry

**Figure 3:**
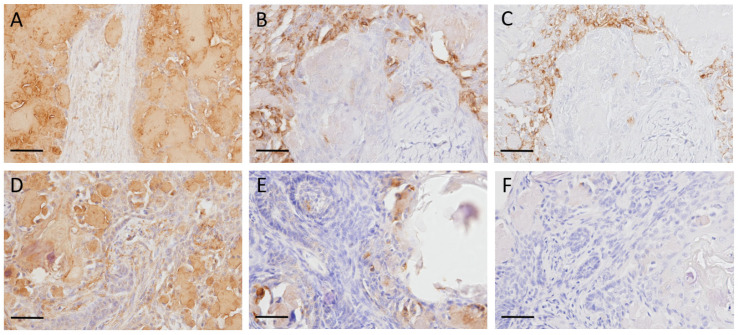
Immunohistochemistry of amyloid-producing ameloblastoma (APAB) in dogs and cats. (**A**–**C**) APAB; dog No. 3. Amyloid deposits were strongly positive to N-terminal region of ameloblastin (**A**), weakly positive to odontogenic ameloblast-associated protein (**B**), and negative to ameloblastin middle region (**C**). (**D**–**F**) APAB; cat No. 2. Amyloid deposits were strongly positive to N-terminal region of ameloblastin (**D**), weakly positive to odontogenic ameloblast-associated protein (**E**), and negative to ameloblastin middle region (**F**). Scale bars: 400 μm.

In all dog and cat cases, amyloid deposits were strongly positive for the N-terminal region of ameloblastin (Figure 3A,D). In contrast, amyloid deposits were negative for the ameloblastin middle region (Figure 3C,F). As for odontogenic ameloblast-associated protein, the amyloid had negative to partially positive reactions (Figure 3B,E). Tumor cells showed varying degrees of positivity for the N-terminal region of ameloblastin, the middle region of ameloblastin, and odontogenic ameloblast-associated protein.

## 4. Discussion

In this study, mass-spectrometry-based proteomic analysis extracted ameloblastin as a prime candidate for precursor protein of APAB, and immunohistochemistry showed evidence for the existence of ameloblastin in amyloid deposits of both cats and dogs. Several immunohistochemistry-based studies in canines and felines have shown that ameloblastin [17,26,28], sheathlin, and amelogenin [17,28] are present in amyloid deposits but are negative for antibodies to cytokeratins and vimentin [17]. The amyloid of APABs from a cat, dog, and Bengal tiger did not contain odontogenic ameloblast-associated protein-related peptides but rather an ameloblastin-like peptide; an N-terminal residue of ameloblastin was detected from collected amyloid deposits of these APAB cases [29]. This study identified ameloblastin as a major component of amyloid deposits via mass-spectrometry-based semi-quantitative analysis using the Mascot algorithm, while sheathlin and amelogenin were barely detected. These data indicate that ameloblastin is the amyloid precursor protein of APABs in dogs and cats.

In dog and cat cases, ameloblastin N-terminal peptides were frequently detected by mass spectrometry, while the peptides from the middle to C-terminal region were rarely detected. The ameloblastin N-terminal peptides often showed non-tryptic digestion, indicating proteolysis of ameloblastin during amyloid formation in vivo. Immunohistochemistry also showed that amyloid deposits were negative for the middle region of ameloblastin and positive for the N-terminal region of ameloblastin. These results clearly support that the N-terminal region of ameloblastin is involved in amyloid formation. In various types of amyloidosis, protein destabilization associated with proteolysis plays an important role in misfolding and subsequent amyloid formation [30]. Further, also in APAB, it is possible that the N-terminal region of ameloblastin is degraded by some mechanism, resulting in the formation of amyloid. It should be noted that, in this study, we had to use the canine protein database, even for the analysis of feline cases. At the time of writing, the exact sequence data for feline ameloblastin had not been deposited in the National Center for Biotechnology Information or UniProt database. To enhance the accuracy of analysis, it will be necessary to determine the full amino acid sequence of feline ameloblastin and include it in mass spectrometry databases. This will enable more precise and reliable analysis in the future. Based on veterinary medicine reports, APAB is a rare tumor in animals, including cats and dogs. A retrospective survey on 486 cases of canine and feline oral cavity tumors and tumor-like lesions reported that APAB was diagnosed only in one dog [31]. The rarity of APAB makes it challenging to study, as there are limited cases to gather data from. This study, though small in scale, was conducted on five cases divided into two species, suggesting that the N-terminal region of ameloblastin is involved in the formation of amyloid in APABs in cats and dogs. Accurate identification of precursor protein in APAB will provide new insight into underlying causes of APAB in cats and dogs. Further studies in this area are necessary to be precise about the detailed mechanisms of amyloidogenesis of ameloblastin.

## 5. Conclusions

This study suggests that ameloblastin is the amyloid precursor protein of APABs in dogs and cats, and the N-terminal region may be involved in the amyloidogenesis of ameloblastin, which highlights the importance of additional investigation in this field of study.

## Figures and Tables

**Table 1:** Case information for dogs and cats included in the study.

Case No.	Breed	Age, years	Sex	Region
Dog No. 1	Toy poodle	10	Female	Left mandibular gingival mass
Dog No.2	Shih-Tzu	Unknown	Female	Left mandibular mass
Dog No.3	Miniature schnauzer	11	Male	Right mandibular mass
Cat No.1	Mix	8	Female	Right maxillary gingival mass
Cat No.2	Unknown	18	Female	Left maxillary mass

**Table 2:** Proteins recognized from microdissected amyloid deposits. Each number refers to the mean Mascot’s probability-based score. ApoA-IV, apolipoprotein A-IV; ApoA-I, apolipoprotein A-I. Lists of full detected proteins are presented in Tables S1–S5.

Case	Ameloblastin	Keratin 5	Keratin 14	ApoA-I	ApoA-IV	Vitronectin	Clusterin
Dog No.1	844	838	1274	-	65	38	-
Dog No.2	795	386	408	57	-	-	-
Dog No.3	735	822	1333	-	51	197	124
Cat No.1	105	-	-	-	-	95	-
Cat No.2	112	399	578	-	-	-	-

## Data Availability

The raw data of the results acquired in this study are available on request from the corresponding author.

## Supplementary Materials

The following supporting information can be downloaded at: https://www.mdpi.com/article/10.3390/vetsci10020166/s1, Figure S1: A Pairwise Sequence Alignment of ameloblastin; Tables S1–S5: Summary of proteins detected by LC/MS/MS.
