# Supplementary material for: Identification of Ameloblastin as an Amyloid Precursor Protein of Amyloid-Producing Ameloblastoma in Dogs and Cats
Source: Vet Sci. 2023 Feb 20;10(2):166. doi: 10.3390/vetsci10020166 (PMC9963920; doi:10.3390/vetsci10020166)
Supplement: Supplementary file 1 [file vetsci-10-00166-s001.zip › vetsci-2206619-supplementary.pdf]

veterinary sciences

MDPI

Supplementary Information for

Identification of ameloblastin as an amyloid precursor protein of amyloid-producing amelo-blastoma in dogs and cats

Niki Sedghi Masoud, Susumu Iwaide, Yoshiyuki Itoh, Miki Hisada, Tomoyuki Harada, Tomoaki Murakami*

This file includes:

Tables S1 to S5

Figure S1

Table S1. Summary of proteins detected by LC/MS/MS of left mandibular gingiva amyloid deposit in dog number one

Rank	Accession	Description	Num. of significant matches	Num. of significant sequences	Score	emPAI*
1	NP_001240670.1	Keratin 14	44	18	1274	2.18
2	XP_548099.3	Keratin, type I cytoskeletal 42	14	8	152	0.7
**3**	**XP_539304.3**	**Ameloblastin**	**24**	**7**	**844**	**0.89**
4	NP_001332964.1	Keratin, type II cytoskeletal 5	41	18	838	1.76
5	XP_003433560.1	Keratin, type II cytoskeletal 6A	17	10	283	0.69
6	XP_543651.2	Keratin, type II cuticular Hb2	3	2	48	0.12
7	XP_543796.1	Histone H2A.J	8	2	235	0.92
8	XP_003639664.1	Histone H2A type 2-C	4	2	91	0.54
9	XP_532744.1	Cationic trypsin-like	6	1	144	0.13
10	NP_001003090.1	Collagen alpha-1(I) chain precursor	2	1	75	0.02
11	XP_546510.3	Apolipoprotein A-IV	5	3	65	0.24
12	XP_013966037.1	uncharacterized protein LOC100856160	2	1	65	0.15
13	NP_001003349.2	Actin, cytoplasmic 2	2	1	51	0.08
14	XP_539002.2	Collagen alpha-1(XII) chain isoform X2	1	1	41	0.01
15	XP_854040.1	Vitronectin	1	1	38	0.06
16	XP_013966933.1	Late histone H2B.L4	1	1	35	0.2

Ameloblastin is shown in bold. *emPAI, exponentially modified protein abundance index.

Table S2. Summary of proteins detected by LC/MS/MS of left mandibular amyloid Deposits in dog number two

Rank	Accession	Description	Num. of significant matches	Num. of significant sequences	Score	emPAI*
**1**	**XP_539304.3**	**Ameloblastin**	**26**	**8**	**795**	**1.18**
2	NP_001240670.1	Keratin 14	16	9	408	0.84
3	NP_001332964.1	Keratin, type II cytoskeletal 5	17	10	386	0.66
4	NP_001003026.1	Serum albumin precursor	15	8	256	0.52
5	NP_001003349.2	Actin, cytoplasmic 2	17	8	189	0.98
6	XP_005618896.1	Actin, alpha skeletal muscle	9	5	111	0.46
7	XP_544346.1	Beta-actin-like protein 2	3	2	47	0.16
8	XP_532744.1	Cationic trypsin-like	8	2	124	0.27
9	XP_005636600.1	immunoglobulin lambda-1 light chain isoform X34	2	1	78	0.13
10	XP_013966037.1	uncharacterized protein LOC100856160	3	2	78	0.31
11	XP_540286.2	Histone H2A type 2-A	2	1	67	0.24
12	XP_013968873.1	Apolipoprotein A-I	3	2	57	0.23
13	NP_001003090.1	Collagen alpha-1(I) chain precursor	1	1	44	0.02
14	NP_001184045.1	Elongation factor 1-alpha 1	1	1	42	0.07
15	NP_001003187.1	Collagen alpha-2(I) chain precursor	2	2	38	0.05

16	XP_013962533.1	Transcription factor COE4	1	1	33	0.05
17	NP_001159360.1	Serpin H1 precursor	1	1	33	0.07
18	XP_022272121.1	LOW QUALITY PROTEIN: tRNA (uracil(54)-C(5))-methyltransferase homolog	1	1	27	0.05
19	XP_534537.3	Serine/threonine-protein kinase LATS2	1	1	23	0.03

Ameloblastin is shown in bold. *emPAI, exponentially modified protein abundance index.

Table S3. Summary of proteins detected by LC/MS/MS of a cystic mass in right mandible amyloid Deposits in dog in number three

Rank	Accession	Description	Num. of significant matches	Num. of significant sequences	Score	emPAI*
1	NP_001240670.1	Keratin 14	43	19	1333	2.59
2	NP_001257812.1	Hemoglobin subunit beta-like	36	10	1221	13.36
3	NP_001332964.1	Keratin, type II cytoskeletal 5	39	17	822	1.62
4	XP_003433560.1	Keratin, type II cytoskeletal 6A	16	7	241	0.52
5	NP_001257815.1	Hemoglobin subunit alpha-like	29	5	758	5.02
**6**	**XP_539304.3**	**Ameloblastin**	**18**	**3**	**735**	**0.42**
7	XP_543796.1	Histone H2A.J	10	3	411	1.38
8	XP_003639664.1	Histone H2A type 2-C	8	3	205	1.38
9	XP_003639985.1	Gamma-enolase	9	2	232	0.22
10	XP_854040.1	Vitronectin	6	3	197	0.19
11	NP_001003026.1	Serum albumin precursor	6	4	185	0.2
12	NP_001003370.1	Clusterin precursor	2	1	124	0.06
13	NP_001003349.2	Actin, cytoplasmic 2	6	4	111	0.35
14	NP_001003090.1	collagen alpha-1(I) chain Precursor	5	3	83	0.07
15	XP_005636600.1	immunoglobulin lambda-1 Light chain isoform X34	1	1	78	0.13
16	XP_022262838.1	Complement C3	2	1	74	0.04
17	XP_536733.1	Protein DJ-1 isoform X2	2	1	71	0.17
18	XP_532744.1	Cationic trypsin-like	2	1	63	0.13
19	XP_531639.2	ATP synthase subunit beta, mitochondrial	1	1	57	0.06
20	NP_001003187.1	collagen alpha-2(I) chain precursor	2	2	51	0.05
21	XP_546510.3	apolipoprotein A-IV	1	1	51	0.07
22	XP_013966037.1	uncharacterized protein LOC100856160	1	1	35	0.15
23	NP_001003142.2	glyceraldehyde-3-phosphate Dehydrogenase	1	1	33	0.09
24	XP_022260119.1	Collectin-11 isoform X2	1	1	31	0.11
25	XP_013975554.1	prolow-density lipoprotein receptor-related protein 1-like isoform X1	1	1	29	0.01
26	XP_005639594.1	probable G-protein coupled Receptor 156 isoform X1	1	1	19	0.04

Ameloblastin is shown in bold. *emPAI, exponentially modified protein abundance index.

Table S4. Summary of proteins detected by LC/MS/MS of right maxillary gingiva amyloid Deposits in cat number one

Rank	Accession	Description	Num. of significant matches	Num. of significant sequences	Score	emPAI
1	NP_001257812.1	hemoglobin subunit beta-like	12	4	290	1.14
2	NP_001003392.1	keratin, type II cytoskeletal 1	7	4	190	0.22
3	XP_532744.1	cationic trypsin-like	7	2	136	0.27
4	XP_539304.3	ameloblastin	3	2	105	0.15

5	XP_854040.1	vitronectin	2	1	95	0.06
6	XP_013972063.1	keratin, type I cytoskeletal 10 isoform X1	5	4	91	0.23
7	XP_542374.3	hemoglobin subunit epsilon	2	1	80	0.21
8	XP_013966596.1	shugoshin 2	1	1	42	0.02
9	NP_001273789.1	carnitine O-palmitoyltransferase 1, liver isoform	1	1	35	0.04
10	XP_022263087.1	mucin-16	1	1	25	0.01

Ameloblastin is shown in bold. *emPAI, exponentially modified protein abundance index.

Table S5. Summary of proteins detected by LC/MS/MS of left maxillary mass amyloid Deposits in cat number two

Rank	Accession	Description	Num. of significant matches	Num. of significant sequences	Score	emPAI
1	NP_001240670.1	keratin 14	26	15	578	1.65
2	XP_548099.3	keratin, type I cytoskeletal 42	17	8	337	0.6
3	XP_548107.2	keratin, type I cuticular Ha6	4	3	58	0.2
4	XP_003433560.1	keratin, type II cytoskeletal 6A	25	14	553	1.09
5	NP_001332964.1	keratin, type II cytoskeletal 5	23	13	399	0.93
6	NP_001003392.1	keratin, type II cytoskeletal 1	7	5	85	0.28
7	NP_001182774.2	actin, cytoplasmic 1	11	6	181	0.7
8	XP_532744.1	cationic trypsin-like	9	3	150	0.43
9	XP_539304.3	ameloblastin	3	2	112	0.15
10	NP_001003026.1	serum albumin precursor	2	1	96	0.05
11	XP_013966037.1	uncharacterized protein LOC100856160	2	1	79	0.15
12	XP_540286.2	histone H2A type 2-A	2	1	76	0.24
13	XP_548841.3	matrix-remodeling-associated protein 5	1	1	34	0.01
14	XP_003432104.1	anionic trypsin	1	1	33	0.13
15	XP_013965707.1	LOW QUALITY PROTEIN: golgin subfamily B member 1	2	1	31	0.01
16	XP_539321.2	histone H2B type 3-B	1	1	29	0.24
17	XP_022271308.1	amelogenin, X isoform	1	1	28	0.14
18	XP_854395.1	endonuclease 8-like 1 isoform X1	1	1	26	0.08
19	XP_539158.2	metastasis suppressor protein 1 isoform X7	1	1	24	0.04
20	XP_013970744.1	dynein heavy chain 14, axonemal isoform X1	1	1	20	0.01

Ameloblastin is shown in bold. *emPAI, exponentially modified protein abundance index.

Supplementary Figure S1. a Pairwise Sequence Alignment of ameloblastin in dog (NCBI accession: XP_539304.3) and cat (NCBI accession: XP_044912677) is shown. The predicted signal sequence of ameloblastin in dogs is underlined.

```
XP_044912677.      1 MVYDSESHRLYTVVSSGTEQKDNSATYPTHYSETLLRVRAGIKGHKAYPS     50

XP_539304.3        1 --------------------------------------------------      0

XP_044912677.     51 KTVLVKWVYNGYHLAGNNPEEKQMNHHFHIQSKQVLLGKLPRTAVSVAHT    100

XP_539304.3        1 --------------------------------------------------      0

XP_044912677.    101 SAINSLVCKGSMQIYSRSVQGSGAGRAGPSLPPGRSLRTTTPLLALALGA    150

XP_539304.3        1 --------------------------------------------------      0

XP_044912677.    151 EGQRFLRTLQALLSPRKLTDRHEWITTEKCLPEAGTTLNEQDECGALECV    200

XP_539304.3        1 --------------------------------------------------      0

XP_044912677.    201 KAASELYSPPAGEVTEINEALEENPALVNKFCYQDVKIPLSKMKDLILIF    250
                                                     ...:||||.|||||:||.
XP_539304.3        1 --------------------------------MSALKIPLFKMKDLVLIL     18

XP_044912677.    251 CLLEMSSAVPMFPQQPGTPGMASLSLETMRQLGSLQGLNMLSQYSRFGFG    300
                     |||:||||||:|||||||||||||||||||||||||||||||||||||||
XP_539304.3       19 CLLKMSSAVPVFPQQPGTPGMASLSLETMRQLGSLQGLNMLSQYSRFGFG     68

XP_044912677.    301 KSFNSLWMHGLLPPHSSFPWMRPREHETQQYEYSLPVHPPPLPSQPSLQP    350
                     ||||||||||||||||||||||||||||||||||||||||||||||||||
XP_539304.3       69 KSFNSLWMHGLLPPHSSFPWMRPREHETQQYEYSLPVHPPPLPSQPSLQP    118

XP_044912677.    351 QQPGQKPFLQPTIVTAIQDAAQKGGPHPPVYQGQPPLQQAEGPMLEQQVA    400
                     ||||||||||..|||.|||.|||.|..||||||||||||.||||||||||
XP_539304.3      119 QQPGQKPFLQSAIVTDIQDTAQKRGTQPPVYQGQPPLQQTEGPMLEQQVA    168

XP_044912677.    401 PSDKPPKAELPGMDFADPQGPSVFQIARLISRGPMPQNKPSPLYPGIFYM    450
                     ||||||||||||||||:|||||||||||||||||||||||||||||||||
XP_539304.3      169 PSDKPPKAELPGMDFAEPQGPSVFQIARLISRGPMPQNKPSPLYPGIFYM    218
```

(In XP_539304.3, the underlined signal sequence is MSALKIPLFKMKDLVLIL CLLKMSSA.)

```
XP_044912677.     451 SYGANQLNAPARLGIMSSEEMAGGRGGPMAYGAMFPGFGGMRPNLGGIPH    500
                      ||||||||||.|||||||||||||||.||||||||||||||||||||:||
XP_539304.3       219 SYGANQLNAPGRLGIMSSEEMAGGRGSPMAYGAMFPGFGGMRPNLGGMPH    268

XP_044912677.     501 NPAMGGDFTLEFDTPVAGTKGPEKGEGGAQGSPRPDVNPADPENPALLPE    550
                      ||.||||||||||:||||||||||||||||||||.||||||:|||||||.|
XP_539304.3       269 NPGMGGDFTLEFDSPVAGTKGPEKGEGGAQGSPMPDVNPANPENPALLTE    318

XP_044912677.     551 VAPGALGGLLAHPKGNDPSLARGPAGQSGGPPRVTPAEADPLMTPGLADI    600
                      :|||||||||||||.||||||||||||||||||||||:|||||||.||||
XP_539304.3       319 LAPGALGGLLAHPKDNDPSLARGPAGQSGGPPRVTPADADPLMTPELADI    368

XP_044912677.     601 YETYGADVTTPLEETPTDTTAIPDTQQTSMPENKAQQPQIMHDVWHFQEP    650
                      ||||||||||||||||||||.|||||||.||||||||||||||.||||||
XP_539304.3       369 YETYGADVTTPLEETPTDTTVIPDTQQTLMPENKAQQPQIMHDGWHFQEP    418
```
